# Region-specific depletion of synaptic mitochondria in the brains of patients with Alzheimer’s disease

**DOI:** 10.1007/s00401-018-1903-2

**Published:** 2018-09-06

**Authors:** Eleanor K. Pickett, Jamie Rose, Caoimhe McCrory, Chris-Anne McKenzie, Declan King, Colin Smith, Thomas H. Gillingwater, Christopher M. Henstridge, Tara L. Spires-Jones

**Affiliations:** 1 Centre for Discovery Brain Sciences, UK Dementia Research Institute, The University of Edinburgh, 1 George Square, Edinburgh, EH8 9JZ UK; 2 Centre for Clinical Brain Sciences, Edinburgh Brain and Tissue Bank, The University of Edinburgh, Edinburgh, UK

**Keywords:** Alzheimer’s disease, Synapses, Mitochondria, Electron microscopy

## Abstract

Of all of the neuropathological changes observed in Alzheimer’s disease (AD), the loss of synapses correlates most strongly with cognitive decline. The precise mechanisms of synapse degeneration in AD remain unclear, although strong evidence indicates that pathological forms of both amyloid beta and tau contribute to synaptic dysfunction and loss. Synaptic mitochondria play a potentially important role in synapse degeneration in AD. Many studies in model systems indicate that amyloid beta and tau both impair mitochondrial function and impair transport of mitochondria to synapses. To date, much less is known about whether synaptic mitochondria are affected in human AD brain. Here, we used transmission electron microscopy to examine synapses and synaptic mitochondria in two cortical regions (BA41/42 and BA46) from eight AD and nine control cases. In this study, we observed 3000 synapses and find region-specific differences in synaptic mitochondria in AD cases compared to controls. In BA41/42, we observe a fourfold reduction in the proportion of presynaptic terminals that contain multiple mitochondria profiles in AD. We also observe ultrastructural changes including abnormal mitochondrial morphology, the presence of multivesicular bodies in synapses, and reduced synapse apposition length near plaques in AD. Together, our data show region-specific changes in synaptic mitochondria in AD and support the idea that the transport of mitochondria to presynaptic terminals or synaptic mitochondrial dynamics may be altered in AD.

**Electronic supplementary material:**

The online version of this article (10.1007/s00401-018-1903-2) contains supplementary material, which is available to authorized users.

## Introduction

Alzheimer’s disease (AD) is the most common cause of dementia affecting around 30 million people worldwide. There are currently no disease-modifying treatments for AD, making understanding the underlying mechanisms of neurodegeneration a high research priority. Pathologically, the disease is defined by brain atrophy and the accumulation of amyloid beta in extracellular plaques and tau in neurofibrillary tangles [54]. The brain atrophy comprises loss of neurons, white matter and synapses.

Synapse loss correlates strongly with cognitive decline in AD when measured by counting synaptic profiles with electron microscopy (EM) or by measuring synaptic protein levels [10, 11, 60]. Both Aβ and tau contribute to synapse dysfunction and degeneration in AD model systems and are observed in synapses in human AD brain [31, 40, 46, 47, 55, 63]. However, the causes of synapse dysfunction and degeneration in the human brain remain largely unknown. Synaptic mitochondria are potentially important players in synapse degeneration in AD brain. Damage to synaptic mitochondria or failure to transport enough mitochondria to synapses could both impair function and lead to synapse collapse.

The role of mitochondria in metabolism is crucial for providing the necessary energy required for neurotransmitter release at the presynapse [62]. ATP generation is mediated through the electron transport chain (ETC), consisting of five protein complexes undergoing sequential redox reactions, which culminate in the production of ATP. Importantly, recent work shows that synaptic mitochondria have distinct morphologies and proteomic profiles compared to non-synaptic mitochondria, which may make synapses particularly vulnerable to degeneration [20]. In AD models, Aβ preferentially blocks complex IV of the ETC [22, 30], whereas tau impairs complex I [8]. By targeting different components of the same system, Aβ and tau amplify one another’s toxic effects [27, 51].

Since the synapse is a site of high-energy demand, it is necessary for mitochondria to be trafficked to this location. Tau plays a crucial role in binding and stabilising microtubules required for this anterograde transport of mitochondria [15]. It has been suggested that pathological tau may interfere with this trafficking process resulting in impaired anterograde transport of cargo [28, 33]. Both in vitro and in vivo models have shown that the overexpression of tau inhibits anterograde mitochondrial transport and disrupts mitochondrial distribution in neurites, resulting in perinuclear clumping in the soma [32, 57]. In addition to tau-associated transport deficits, oligomeric Aβ has also been implicated in the impairment of mitochondrial movements in hippocampal cultures [5, 9, 52, 53]. Considering that both tau and Aβ have been implicated in disrupted anterograde mitochondrial transport, depletion of mitochondria at the synapse may be a synergistic mechanism contributing to synaptotoxicity. Data from human AD brain demonstrate that mitochondria accumulate in dystrophic neurites [56], which implies that they may be stuck in dystrophies and prevented from reaching synaptic terminals. To formally test the hypothesis that the presence of mitochondria in synaptic terminals is altered in AD, we used transmission EM to quantify synaptic mitochondria in two cortical regions—BA46 and BA41/42. Furthermore, we examined ultrastructural features of synapses in these brain regions. We find a reduction in the percentage of presynaptic terminals containing multiple mitochondria in BA41/42 of AD patients compared to control subjects, and we observe abnormal mitochondrial morphology in synapses in AD but not control cases. Our results indicate that synaptic mitochondria are affected in a region-specific manner in Alzheimer’s disease, which may impair synaptic function and cognition.

## Materials and methods

### Post-mortem analysis

Tissue from eight clinically and pathologically diagnosed Alzheimer’s disease donors and nine control donors were used for this study (details in Online Resource 1). Use of human tissue for post-mortem studies has been reviewed and approved by the Edinburgh Brain Bank ethics committee and the ACCORD medical research ethics committee (approval HV-15-016; ACCORD is the Academic and Clinical Central Office for Research and Development, a joint office of the University of Edinburgh and NHS Lothian). The Edinburgh Brain Bank is a Medical Research Council funded facility with research ethics committee (REC) approval 16/ES/0084.

### Tissue preparation

At post-mortem, the brain was removed and cut into coronal slices. Regions of interest were then dissected from each coronal slice. Samples from one hemisphere were dissected into smaller segments and processed for electron microscopy as described previously [29]. Fresh post-mortem samples from BA46 (dorsolateral prefrontal cortex) and BA41/42 (anterior/posterior transverse temporal cortex) were trimmed into small cortical blocks containing the six cortical layers and fixed in 4% paraformaldehyde and 2.5% glutaraldehyde in 0.1 M phosphate buffer (PB) for 48 h. Fixed tissue blocks were washed twice in 0.1 M PB and were exposed to osmium tetroxide (1% in 0.1 M PB) for 30 min (protected from light). Samples were washed twice for 15 min in 0.1 M PB and three times in previously boiled ddH_2_O. Tissue blocks were then dehydrated for 15 min in 50% ethanol followed by exposure to 1% uranyl acetate in 70% ethanol for 40 min in the dark. Samples were further dehydrated in an ascending series of ethanol (95%, 100%, 100%) and propylene oxide before storage in Durcupan resin overnight at room temperature. Samples were embedded in Durcupan resin and allowed to polymerise for 48 h at 60 °C.

### Tissue sectioning and imaging

**Fig. 1:**
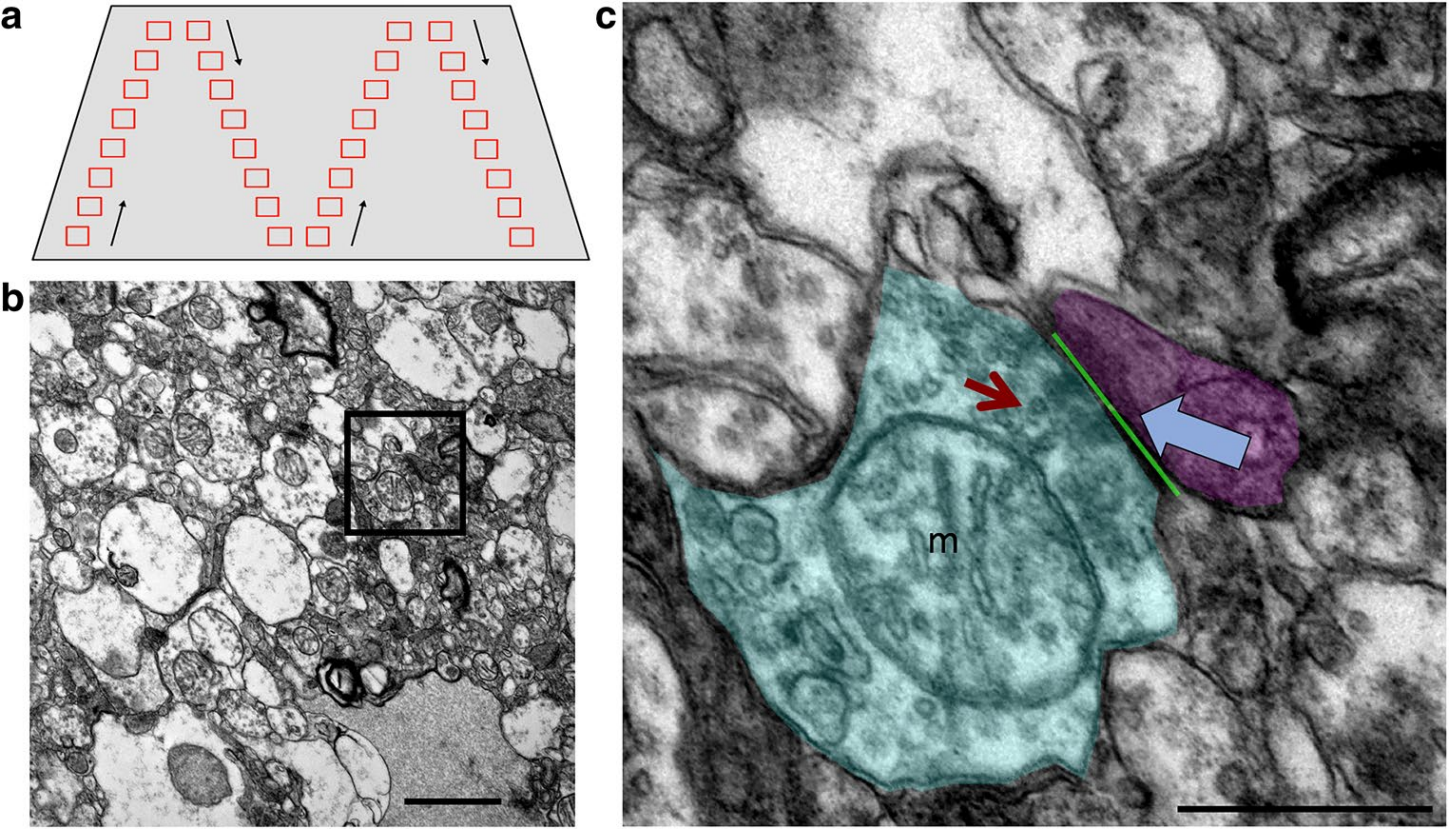
EM Sampling. TEM images were taken throughout the neuropil in a systematic fashion to ensure sampling from the entire tissue block face without repeated sampling or bias (**a**). Images were taken at ×6000 magnification (**b**). Individual synapses (inset in **b**, **c**) were identified by the presence of at least three presynaptic vesicles (red arrow) and a clearly identifiable, electron-dense post-synaptic density (blue arrow). The presence of mitochondria (m) in the pre- (shaded cyan) or post-(shaded magenta) synaptic terminals was recorded and the length of the PSD opposed to the presynaptic active zone was measured (green line). Scale bars represent 1 μm (**b**), 500 nm (**c**)

Resin-embedded tissue blocks were cut into 70-nm-thick sections using an ultracut microtome (Leica) equipped with a Jumbo Histo Diamond Knife (Diatome, Hatfield, PA, USA) and collected onto copper formvar-coated grids. Grids were stained with lead citrate in a CO_2_-free environment for 2 min before imaging on a JEOL JEM-1400 Plus transmission electron microscope (TEM). For synapse analysis, an average of 50 images per case was taken at 6000× magnification in a systematic, random fashion from BA46 and BA41/42 (Fig. 1). Exclusion criteria for sampling images included the presence of a nucleus in the entire field of view, no synapses present in the field of view or the presence of an Aβ plaque or NFT in the entire field of view. TEM images were coded for blind analysis. Synapses were defined by a presynaptic terminal containing at least three synaptic vesicles adjacent to an electron-dense post-synaptic density. Mitochondria were defined by the presence of internal cristae and a defined outer membrane. 100 synapses per case for each brain region (where available, please see Online Resource 1) were analysed for a total of 3000 synapses analysed for the presence of mitochondria (individual or multiple) in the pre- or post-synapse and any unusual features in the synapse noted (abnormal mitochondrial morphology, presence of multivesicular bodies, or presence of fibrils). The length of the post-synaptic density opposing the presynaptic terminal was also measured (apposition length). Extra blocks from temporal, occipital, and frontal cortex were examined for plaques in three AD cases and all plaque-associated synapses pooled for apposition length measurements (80 synapses near plaques analysed). Single sections were imaged as opposed to reconstructing three-dimensional reconstructions of serial sections to allow imaging of large numbers of synapses. We have previously demonstrated that 2D EM quantification techniques performed on synapses, such as those used in our present study, generate near identical findings to parallel 3D quantification studies [19].

### Statistical analysis

Images were analysed by an experimenter blind to diagnosis. For each variable, a single value was calculated per region per case. Statistics were calculated in SPSS and GraphPad Prism. Normality of the data was tested with a Shapiro–Wilk test. Normally, distributed data (apposition length, and percentages of pre- and post-synapses containing a single mitochondrion) were analysed by two-way ANOVA and Tukey’s post hoc tests and are shown as mean and standard errors. Non-normally distributed data (percentages of pre- and post-synapses containing more than one mitochondria and percentage of post-synapses containing multivesicular bodies) were analysed with non-parametric Kruskal–Wallis tests and are shown as median and interquartile ranges.

## Results

### Presynaptic terminals in AD superior temporal gyrus have fewer mitochondria than controls

To test the hypothesis that mitochondrial localisation in synapses is affected in AD, synapses were analysed from AD and control brain samples using transmission electron microscopy (Fig. 1). Raw EM images are freely available from the University of Edinburgh Data Repository DataShare at 10.7488/ds/2417.

**Fig. 2:**
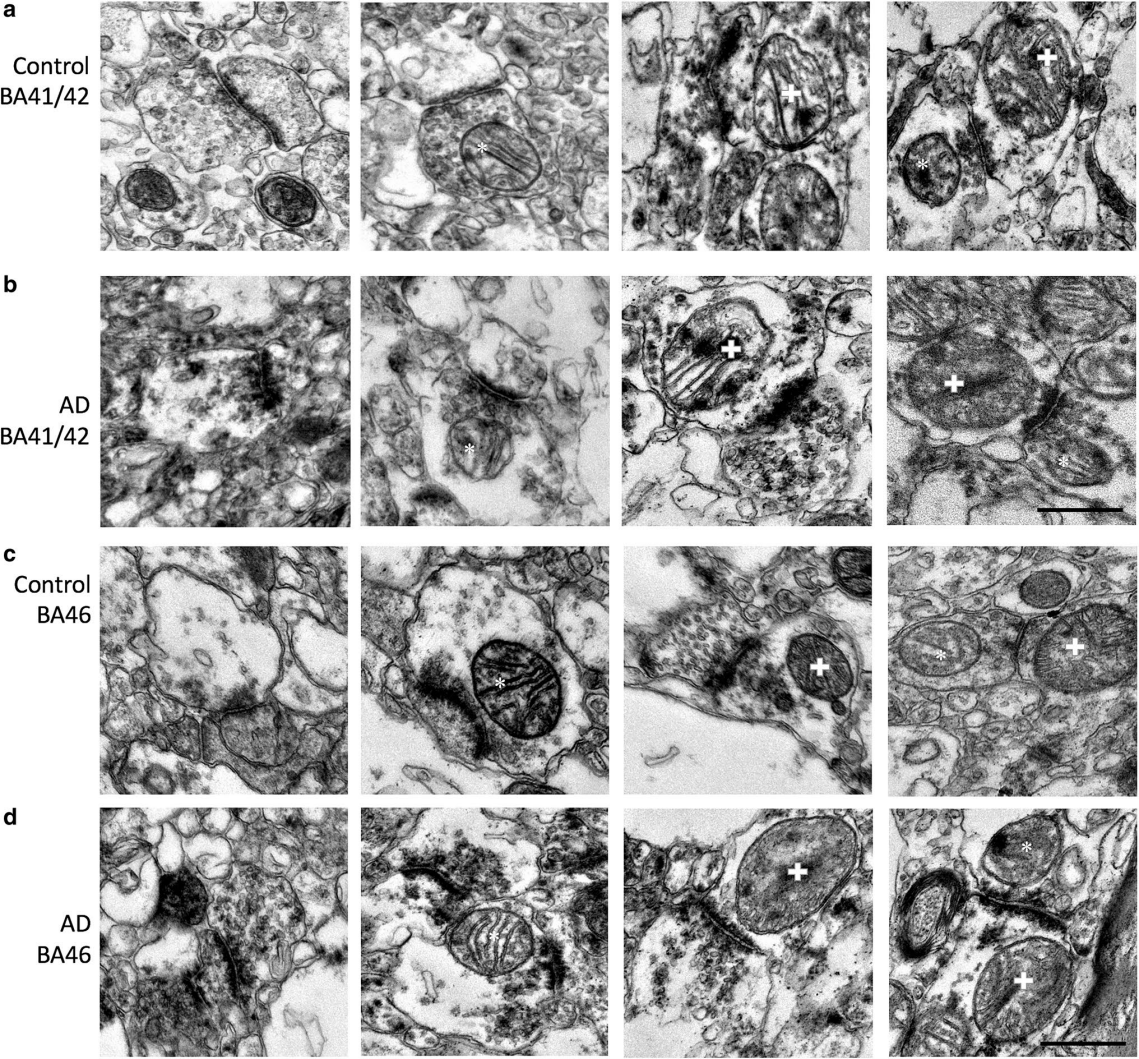
Examples of synapses and synaptic mitochondria. Mitochondria were observed both in presynaptic terminals (asterisks) and post-synaptic terminals (crosses) in control and AD subjects in BA41/42 (**a**, **b**) and BA46 (**c**, **d**). Scale bar 500 nm

**Fig. 3:**
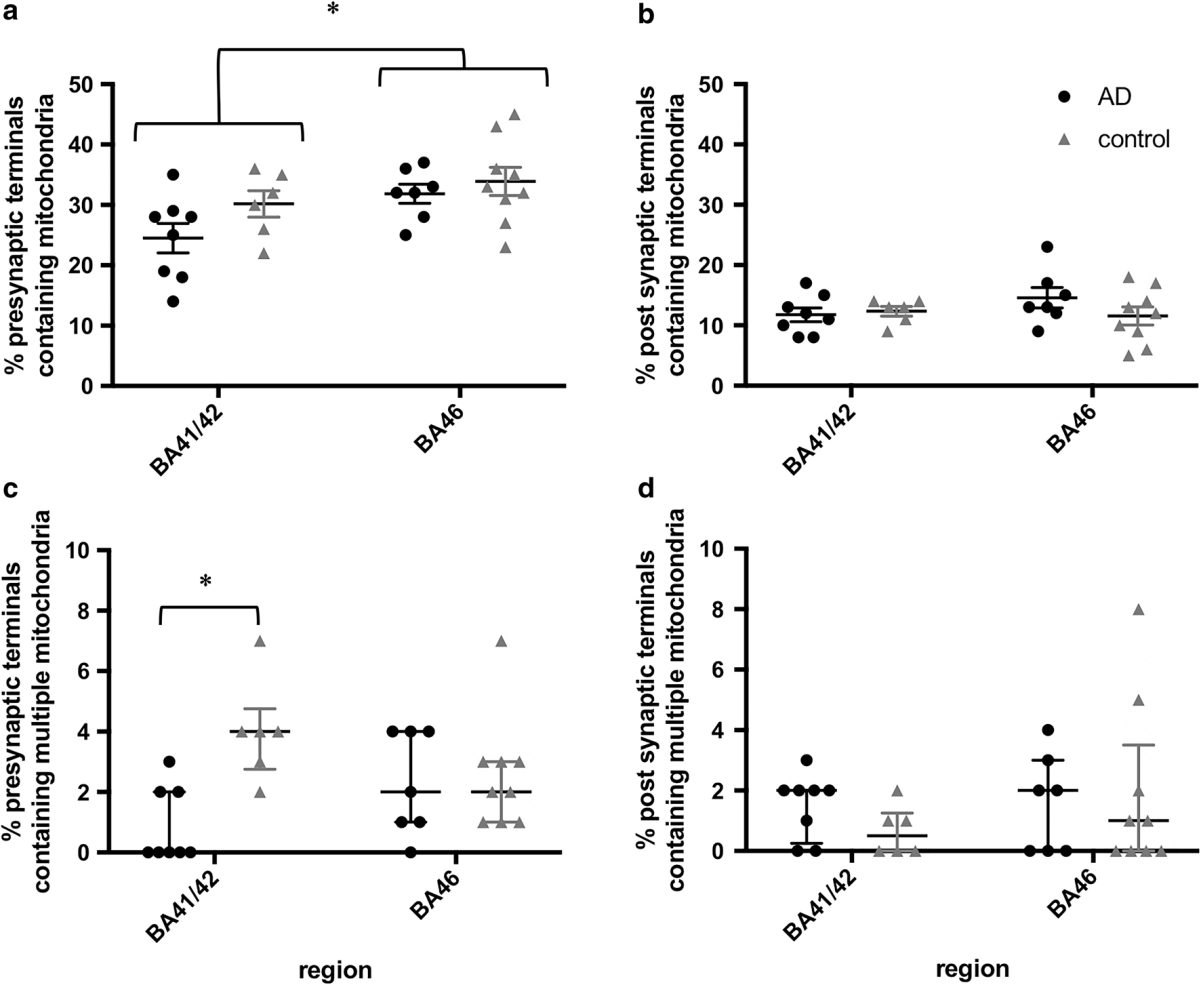
Region-specific depletion of presynaptic mitochondria in AD. The percentage of presynaptic mitochondria is lower in BA41/42 than in BA46 (**a**, asterisk: two-way ANOVA effect of *F* (1, 26) = 5.965, *p* = 0.022). There were no differences in region or disease condition in the percentage of post-synaptic terminals containing mitochondria (**b**). Presynaptic terminals containing more than one mitochondria were over four times less common in presynaptic terminals of AD BA41/42 (**c**, asterisk: independent samples Kruskal–Wallis test, *p* = 0.004). Each data point shows the percentage for an individual case. Data are represented as mean and standard errors in **a**, **b** and medians with interquartile ranges (**c**, **d**)

**Fig. 4:**
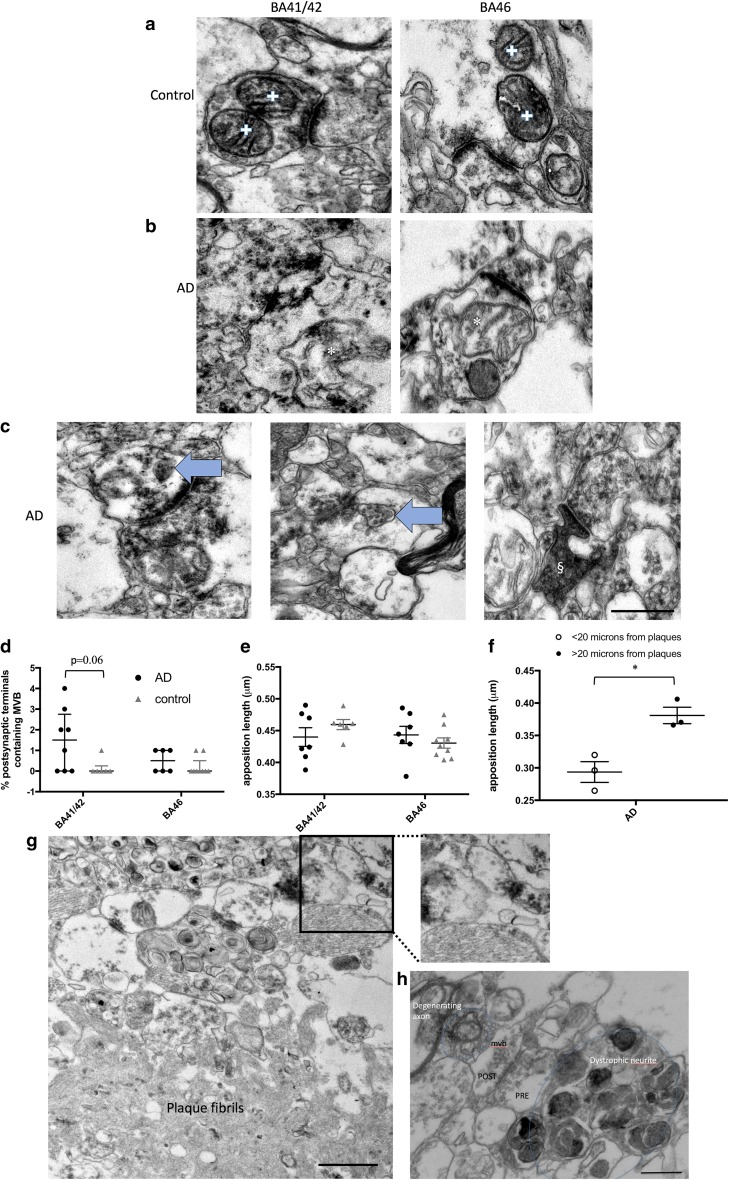
Changes in synaptic morphology in AD. In control cases, multiple mitochondrial profiles in individual pre-synapses were observed (**a**, crosses), while in AD cases, mitochondria with irregular profiles were observed in synapses (**b**, asterisks). Multivesicular bodies (MVB, arrows, **c**) were observed in a subset of post-synapses, and occasional dark degenerating spines (§, **c**) were observed in AD cortex. MVB appeared most often in AD BA41/42 synapses where there was a trend to increase compared to control (**d**, Kruskal–Wallis, *p* = 0.06). Apposition length was unchanged in AD vs. controls in BA41/42 or BA46 (**e**). When more blocks were examined from temporal, frontal and occipital regions to find synapses near plaques (**f**, **g**) and the data combined, we observe significantly decreased apposition length in synapses near plaques compared to those far from plaques (**f**, asterisk: unpaired *t* test with Welch correction, *t* = 4.28, *p* = 0.01). **g** An example of a small synapse near a plaque. **h** More detail around a plaque including a synapses (with pre- and post-synaptic terminals labelled, the post-synapse contains a MVB), a dystrophic neurite, and a degenerating axon. Data are shown as median with interquartile range. Scale bars represent 500 nm (**a**–**c**, inset **g**, **h**); 1000 nm (large panel **g**)

We systematically sampled cortex to analyse synaptic mitochondria in asymmetric synapses in two brain regions, superior temporal gyrus (BA41/42) and dorsolateral prefrontal cortex (BA46). These brain regions play an important role in memory encoding and recognition (BA46) and auditory working memory (BA41/42), which are disrupted in AD [2, 58], and by the end stages of disease, both of these regions have substantial plaque and tangle pathology [54]. We observed mitochondria in a subset of pre- and post-synapses in both regions of AD and control brains (Fig. 2). Two- to threefold more presynaptic terminals contained mitochondria (ranging from 14 to 37% in AD cases and 22–45% in controls) than post-synaptic terminals (ranging from 8 to 23% in AD cases and 5–17% in controls). In both AD and control cases, the higher percentage in pre-synapses than in post-synapses was significant (two-way ANOVA effect of pre vs. post in AD *F*(1, 26) = 68.84, *p* < 0.001; in control *F*(1, 26) = 105.3, *p* < 0.001). Interestingly, there was a trend towards a region effect in the difference between pre- and post-synaptic mitochondria only in AD (two-way ANOVA effect of region in AD *F*(1, 26) = 7.91, *p* = 0.093; control *F*(1, 26) = 0.57, *p* = 0.46). The presence of mitochondria in presynaptic terminals was lower in BA41/42 than in BA46 (Fig. 3a), whereas the percentage of post-synaptic profiles containing mitochondria did not differ by region or with disease (Fig. 3b). A small fraction (0–8%) of synaptic terminals contained more than one mitochondrial profile. In BA41/42, AD cases had over fourfold fewer presynaptic terminals with multiple mitochondrial profiles than control synapses from the same region (Figs. 3c, 4a). The percentage of pre-synapses containing multiple mitochondria was not different between AD and control in BA46, indicating that transport of mitochondria to presynaptic terminals may be impaired in a region-specific manner.

To ensure the significant decrease in presynaptic terminals containing multiple mitochondria was not an artefact of post-mortem degradation, we ran correlation analyses to confirm that there is not an association of PMI or brain pH with this outcome measure. None of the measures of mitochondria in synapses correlated with pH or PMI in either AD or control groups. When considering all cases (AD and controls) together, there is a strong correlation (*r* = − 0.76, *p* < 0.001) with disease status and % presynaptic terminals containing multiple mitochondria in BA41/42 as would be expected from our significant difference found with ANOVA.

Multiple mitochondrial profiles in a single presynaptic bouton in the single-section images that we analysed for the 3000 synapses in the study could correspond to multiple mitochondria or to different parts of the same mitochondrion that has curved out of the plane of view. Three-dimensional reconstructions of two pre-synapses with multiple mitochondria show separate mitochondria in presynaptic terminals (Online Resource 2, Online Resource 3).

Along with the localisation of mitochondria to synapses, we examined the ultrastructure of synapses and synaptic mitochondria. We noted occasional abnormal mitochondrial morphology in AD cases (Fig. 4b) which may indicate mitochondrial dysfunction. Further, we observed accumulation of multivesicular bodies in post-synaptic terminals in BA41/42 of AD cases which was rare but exhibited a trend towards significance (Fig. 4c, d). Apposition length was not changed by region or diagnosis (Fig. 4e). Previous data using array tomography indicated synapse shrinkage near plaques [31]. We did not find enough plaques in the BA41/42 and BA46 samples examined in the systematic random fashion for the main study to compare synapses near and far from plaques. However, we screened blocks for plaques from temporal, frontal, and occipital cortices from three of the AD cases to find more plaques to allow investigation of apposition length of synapses near plaques. A total of 80 synapses near plaques and 219 synapses far from plaques in the same blocks were observed across the three AD cases examined. The average apposition length for each case near and far from plaques was calculated and found to be 23% smaller near plaques than far from plaques (Fig. 4f, g).

**Fig. 5:**
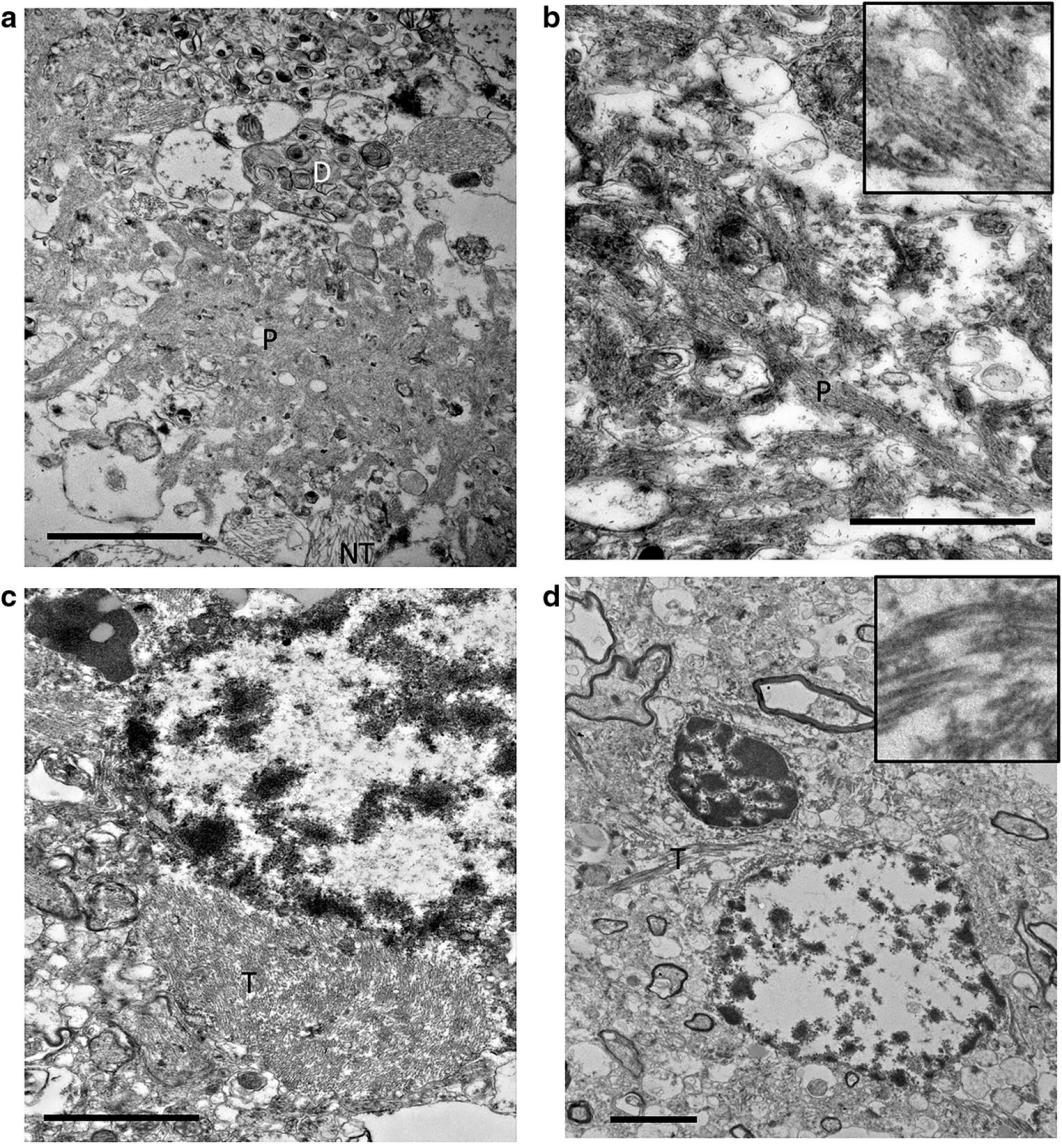
Fibrils in amyloid plaques and neurofibrillary tangles are observed in tissue from Alzheimer’s disease cases with TEM. Aβ plaques (P) surrounded by dystrophic neurites (D) and neuropil threads (NT) are detected in the neuropil from tissue derived from AD cases (**a**, **b**). NFT are observed around the soma of neurons from AD tissue (labeled T in **c**, **d**). Scale bar represents 2 μm in **a**, **c**, and **d**; 1 μm in **b**, inset 500 nm × 500 nm in **b**, 1000 × 1000 nm in **d**

It is also worth noting that of the 3000 synapses systematically sampled in BA41/42 and BA46 and in the 80 synapses near plaques from all regions, we did not find any pre- or post-synaptic terminals containing fibrils, despite the clear appearance of plaque and tangle fibrils in AD cases (Fig. 5). In our previous studies, we detect a substantial proportion of synapses near plaques that are positive for oligomeric Aβ using array tomography [31]. The current ultrastructural data indicate that these oligomers are not yet fibrillar.

## Discussion

Proper synaptic function requires the recruitment of mitochondria to these specialised regions, where energy is in high demand, and elevated levels of calcium are generated in response to synaptic activity. To meet these needs, neuronal synaptic terminals contain a greater number of mitochondria than other cellular regions [3, 36]. Both docked and motile mitochondria are present at presynaptic sites to provide ATP and influence synaptic vesicle release [59]. Mitochondrial disruption at the synapse has been well documented in multiple models of neurodegenerative diseases and commonly appears to be a consequence of increased cellular stress [35]. In the case of Alzheimer’s disease, numerous in vitro and in vivo studies have now reported disruption to the trafficking, dynamics and proteome of these organelles [7, 18, 24, 32, 57].

In recent years, it has been recognised that pathological forms of both Aβ and tau may play a role in these disruptions [34, 50]. In the present study, using post-mortem human brain tissue from individuals with AD pathology and control tissue, a region-specific depletion in the proportion of presynaptic terminals containing multiple mitochondria was observed in the diseased state. This reduction was detected in BA41/42 tissue from individuals with Alzheimer’s disease, whilst BA46 appeared resistant to this loss. The current study suggests a selective vulnerability of BA41/42 synapses to mitochondrial depletion. Temporal cortex has previously been reported to be particularly vulnerable to deficits in complex IV of the mitochondrial respiratory chain in comparison with frontal cortex in individuals with Alzheimer’s disease [39].

A possible explanation for this reduction in mitochondria may be a result of disrupted anterograde transport. Axonal transport defects have been widely reported in culture models of AD, with several studies indicating that the pathological forms of Aβ, APP, PS1 and tau can all affect fast axonal transport [13, 25, 26, 48]. For example, application of Aβ fragments and oligomers in cultured hippocampal neurons have been shown to reduce the proportion of mitochondria capable of moving towards the synapse in an NMDA receptor-dependent mechanism [9, 25]. However, such studies utilise non-physiological, higher levels of amyloid beta, which may contribute to the reported deficits. Pathological forms of tau have also been proposed to inhibit anterograde transport via different mechanisms [13, 16, 57]. However, it has been suggested that the reported tau-mediated disruptions may be a consequence of tau overexpression and that tau may only interfere with trafficking when it is present at high levels. Therefore, in the present study we analysed the number of presynaptic mitochondria present in human brain tissue with AD pathology in the absence of exogenous overexpression systems. Taken together, deficits in the recruitment and redistribution of mitochondria to presynaptic terminals in AD may be responsible for the reduced mitochondrial accumulation observed in BA41/42 pre-synapses. Similar alterations in mitochondrial localisation have also been reported in human AD neurons that contain aggregates of misfolded tau, suggesting that soluble forms of tau may have negative consequences on the cellular distribution of mitochondria [32].

An alternative explanation for the observed decrease in the percentage of pre-synapses with multiple mitochondria in BA41/42 could be due to alterations in mitochondrial morphology. Previous studies have reported alterations in the ultrastructure of mitochondria under pathological conditions including swelling of these organelles [6, 43]. In the presence of Aβ, an exacerbation in the opening of the mitochondrial permeability transition pore (mPTP) has been reported [42, 43]. The resulting increase in the permeability of the inner mitochondrial membrane leads to the influx of fluid and an increase in mitochondrial size which has previously been reported in response to Aβ [45]. The presence of larger mitochondria may occupy space within presynaptic sites preventing further docking of mitochondria at this location. Altered fission or fusion of mitochondria in AD could also contribute to our observed change in presynaptic terminals containing multiple mitochondria. There is evidence supporting altered mitochondrial dynamics in many neurodegenerative diseases including AD [4]. A further explanation for a reduction in mitochondria present at presynaptic sites could be accounted for by an increase in mitochondrial turnover by mitophagy. Previous studies in AD patient brains have reported autophagic accumulation of mitochondria suggestive of enhanced mitophagy induction [23, 44].

Under stress conditions such as hypoxia-reoxygenation, mitochondrial uncoupling and complex inhibition, additional morphologies such as donut, cup and blob have been reported in cells, mice, primates and humans [1, 21, 37, 61]. One study in aged Rhesus monkeys suggested that these morphological changes also appear to accompany functional changes; working memory in these monkeys appeared to correlate positively with straight mitochondria and inversely with donut mitochondria in the presynaptic boutons of dorsal lateral prefrontal cortex (dlPFC). It has been suggested that donut mitochondria are markers of early cellular stress [1, 38]; however, these O-shaped and cup-shaped (‘C’ and ‘U’ shaped) organelles have been observed not only in pathological tissue [12] but in healthy tissue also [17]. Whether these forms are present in the Alzheimer’s diseased brain has yet to be ascertained. In this study, we did not perform 3D reconstructions of all of the synapses studied, so we cannot draw firm conclusions about mitochondrial morphology, but we did observe mitochondrial profiles in single sections that had abnormal morphology.

The maintenance of a pool of mitochondria at AD synapses from BA46 may reflect the resistance of mitochondria from this brain region to the toxic effects of Aβ and tau. Growing evidence suggests that prefrontal synapses remain relatively intact until later stages of the disease as a result of trophic effects that partially compensate for the early phases of degeneration [41]. Whether this support applies to the maintenance of synaptic mitochondria is not known, but may account for a maintained population in this brain region. However, in the present study, late-stage AD brains were examined; therefore, this compensatory protection from synaptic loss maybe ineffective by this stage of disease. It must also be recognised that the synapses sampled consist of those that remain at the end stages of the disease process. Consequently, these synapses may themselves be more resilient to pathological changes. It could be possible that mitochondrial changes at the synapse may be more visible in moderate stages of the disease where synapses remain prior to extensive degeneration and loss.

Our previous data indicate synapse shrinkage in the immediate vicinity of plaques in BA41/42, and the accumulation of oligomeric Aβ and phosphorylated tau within synapses [31]. Here, we did not observe any change in the length of the PSD opposed to the presynaptic terminal; however, there were not enough plaques in the small EM samples to perform the analogous study to our previous work using the higher throughput array tomography technique. The lack of synapse shrinkage when looking both near and far from plaques is in agreement with a recent study using three-dimensional EM which revealed that many morphological features of remaining synapses in AD transentorhinal cortex remain unchanged despite global loss of synapses [14]. The absence of fibrils in pre- and post-synaptic terminals supports previous work strongly implicating soluble but not fibrillar forms of Aβ and tau in synapse toxicity [47, 55]. In addition to occasional mitochondria with abnormal pathology, we observed multivesicular bodies in a small subset of post-synaptic terminals, which is interesting in light of the role they play in the secretion of Aβ [49].

Together, these data indicate that synaptic mitochondria are reduced in presynaptic terminals in AD in a region-specific manner. The more pronounced effect in presynaptic terminals and a lack of change in the presence of mitochondria in post-synapses support the notion that axonal transport of mitochondria at long distances to synaptic terminals is impaired in vulnerable brain regions in AD.

## Electronic supplementary material

Below is the link to the electronic supplementary material.

Supplementary material 1 (XLSX 13 kb)

Supplementary material 2 (PDF 3906 kb)

This movie shows serial sections through a large presynaptic terminal (pink) that makes synapses with two postsynaptic terminals (light green). The presynaptic terminal contains 3 individual mitochondria (blue, yellow, and dark green), and one of the postsynaptic terminals contains a mitochondrion (turquoise) (MOV 14229 kb)
