# Supplementary material for: Region-specific depletion of synaptic mitochondria in the brains of patients with Alzheimer’s disease
Source: Acta Neuropathol. 2018 Sep 6;136(5):747–57. doi: 10.1007/s00401-018-1903-2 (PMC6208730; doi:10.1007/s00401-018-1903-2)
Supplement: Supplementary file 2 — Supplementary material 2 (PDF 3906 kb) [file 401_2018_1903_MOESM2_ESM.pdf]

Region-specific depletion of synaptic mitochondria in the brains of patients with Alzheimer's disease *Acta Neuropathologica* 2018

Eleanor K. Pickett[1], Jamie Rose,[1] Caoimhe McCrory[1], Chris-Anne McKenzie[2], Declan King[1], Colin Smith[2], Thomas H Gillingwater[1], Christopher M. Henstridge[1], and Tara L. Spires-Jones[1]*

Corresponding Author Email: Tara.Spires-Jones@ed.ac.uk

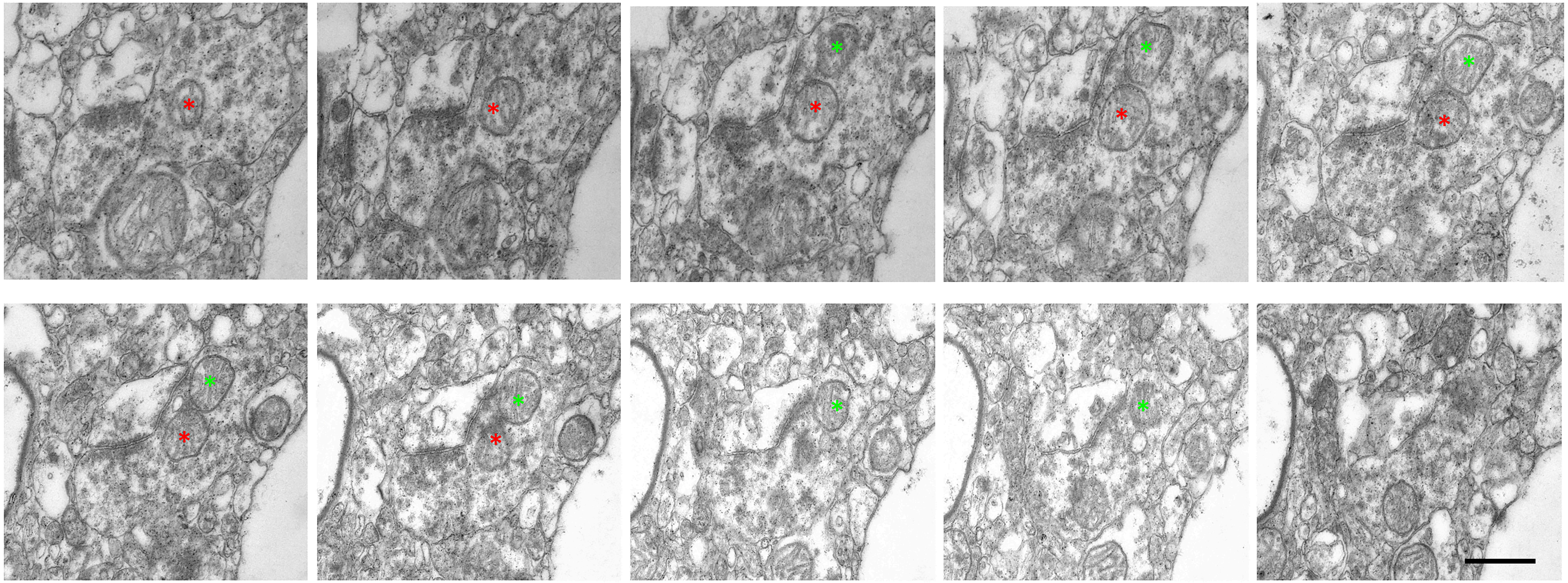

Online Resource 2: Multiple mitochondrial profiles confirmed to correspond to multiple mitochondria in 3D reconstructions. 10 serial 70nm sections show two complete mitochondria (labelled with asterisks) in a presynaptic terminal. Scale bar 500 nm.
